# Regulation of Tyrosinase Gene Expression by Retinoic Acid Pathway in the Pacific Oyster *Crassostrea gigas*

**DOI:** 10.3390/ijms232112840

**Published:** 2022-10-25

**Authors:** Qianqian Jin, Chuncao Huo, Wenhao Yang, Kaidi Jin, Shuai Cai, Yanxin Zheng, Baoyu Huang, Lei Wei, Meiwei Zhang, Yijing Han, Xuekai Zhang, Yaqiong Liu, Xiaotong Wang

**Affiliations:** 1 School of Agriculture, Ludong University, Yantai 264025, China; 2 Changdao Enhancement and Experiment Station, Chinese Academy of Fishery Sciences, Yantai 265800, China

**Keywords:** retinoic acid, retinoic acid receptors, tyrosinase gene, RARES, *Crassostrea gigas*

## Abstract

Retinoic acid (RA) plays important roles in various biological processes in animals. RA signaling is mediated by two types of nuclear receptors, namely retinoic acid receptor (RAR) and retinoid x receptor (RXR), which regulate gene expression by binding to retinoic acid response elements (RAREs) in the promoters of target genes. Here, we explored the effect of all-trans retinoic acid (ATRA) on the Pacific oyster *Crassostera gigas* at the transcriptome level. A total of 586 differentially expressed genes (DEGs) were identified in *C. gigas* upon ATRA treatment, with 309 upregulated and 277 downregulated genes. Bioinformatic analysis revealed that ATRA affects the development, metabolism, reproduction, and immunity of *C. gigas*. Four tyrosinase genes, including *Tyr-6* (LOC105331209), *Tyr-9* (LOC105346503), *Tyr-20* (LOC105330910), and *Tyr-12* (LOC105320007), were upregulated by ATRA according to the transcriptome data and these results were verified by real-time quantitative polymerase chain reaction (RT-qPCR) analysis. In addition, increased expression of *Tyr* (a melanin-related TYR gene in *C. gigas*) and *Tyr-2* were detected after ATRA treatment. The yeast one-hybrid assay revealed the DNA-binding activity of the RA receptors *Cg*RAR and *Cg*RXR, and the interaction of *Cg*RAR with RARE present in the *Tyr-2* promoter. These results provide evidence for the further studies on the role of ATRA and the mechanism of RA receptors in mollusks.

## 1. Introduction

Retinoic acid (RA), a metabolite of vitamin A, plays several key roles in cell differentiation, embryonic development, growth and reproduction, immune function, and organ regeneration in vertebrates [1,2,3]. There are three main isomeric forms of RA, namely all-trans-RA (ATRA), 13-cis-RA (13cRA), and 9-cis-RA (9cRA). Among these, ATRA is the main biologically active isomer [4]. As a ligand of many nuclear receptors, RA is involved in numerous biological processes through the direct or indirect regulation of hundreds of genes, including those related to transcription factors, enzymes, structural proteins, cell surface receptors, neurotransmitters, neuropeptide hormones, and growth factors [5,6].

Earlier RA signaling was considered unique to vertebrates until the enzymes, nuclear receptors, and binding proteins associated with the RA signaling pathway were discovered in invertebrates [7,8]. The identification of RA signaling components implies that RA in invertebrates may have similar functions to those observed in vertebrates. A growing number of studies have further uncovered the role of RA in mollusks. For example, high concentrations of RA can affect shell structure and have toxic effects on eye formation during developmental stages [9]. In *Lymnaea stagnalis*, RA can modify electrical synapses of central neurons and have a potential trophic effect during synaptogenesis [10,11]. In addition, RA appears to induce imposex in marine gastropods via an unknown mechanism [12].

Two classes of receptors, retinoic acid receptor (RAR) and retinoid X receptor (RXR), are responsible for the activation of RA signaling. Three RARs (RARα, RARβ, and RARγ) and three RXRs (RXRα, RXRβ, and RXRγ) of different isoforms have been identified in mammals [13]. Both RARs and RXRs belong to the nuclear receptor superfamily and share a highly conserved DNA-binding domain (DBD) and a relatively conserved ligand-binding domain (LBD) [13,14]. RARs form heterodimers with RXRs after RA perception to regulate gene expression by binding to specific DNA sequences in the promoter region of target genes, which are known as retinoic acid response elements (RAREs) [14,15]. The first RARE identified was a direct repeat (DR) of two (A/G)GGTCA half-site core sequences separated by five base pairs, called DR5 [16]. The vast majority of RAREs identified to date consist of two copies of the (A/G)G(G/T)(G/T)(G/C)A core sequence, organized as DRs and separated by a variable number of nucleotides. Of these, RAREs configured as DRs of the (A/G)G(G/T)TCA core sequence are best known [17].

RA receptors have also been identified in several mollusk species. Two RXR isoforms identified in *Thais clavigera* can form heterodimers with RAR-like proteins and displayed 9c-RA dependent transcriptional activity [18,19]. In *Nucella lapillus*, the retinoic acid receptor *Nl*RAR combines with the RXR to form a heterodimer that specifically binds to RAREs organized in DRs [20]. In *C. gigas*, both RXR and RAR have been isolated [21]. Several studies have revealed that *Cg*RXR not only binds to *Cg*RAR [22], but also acts as a co-receptor to form heterodimers with *Cg*TR [23] and *Cg*PPAR2 [24]. Other studies have shown that *Cg*RXR can combine with DRs, with AGGTCA as the core sequence spaced by 0–5 nucleotides in vitro [25].

Tyrosinase, a member of the type-3 copper protein superfamily, is a complex oxidoreductase comprised of multiple subunits [26,27]. Tyrosinase is widely present in plants, microorganisms, and animals and is involved in a variety of biological processes, including oxygen transport, innate immunity, wound healing, and pigmentation [27,28]. Tyrosinase has a well-defined melanogenic enzyme catalytic activity, and is the rate-limiting enzyme that regulates melanin production [29]. In *C. gigas*, at least 26 genes encode tyrosinase isoforms [30], and these are mainly related to shell formation, prismatic pigmentation, and melanin synthesis [31,32,33].

Our previous study showed that ATRA could activate the expression of the RA receptors *Cg*RAR and *Cg*RXR, and that *Cg*RAR could be found in the nucleus of mammalian cells. Furthermore, *Cg*RAR could combine with *Cg*RXR through the LBD domain. In the current study, transcriptome analysis following ATRA treatment was performed to further investigate the effects of RA in *C. gigas*. Differentially expressed genes (DEGs) between the ATRA and dimethyl sulfoxide (DMSO) control groups were assayed, and we found that the expression of several TYR genes were significantly upregulated after ATRA treatment. Real-time quantitative polymerase chain reaction (RT-qPCR) analysis revealed that the expression of several TYR genes, including *Tyr*, *Tyr-2*, *Tyr-6*, *Tyr-9*, *Tyr-12*, and *Tyr-20*, were significantly upregulated after ATRA treatment. The yeast one-hybrid assay showed that the RA receptors *Cg*RAR and *Cg*RXR bind to the DR0-DR5 sequence, and that *Cg*RAR can bind to the *Tyr-2* promoter.

## 2. Results

### 2.1. Transcriptome Analysis upon ATRA Treatment in the Pacific Oyster

**Figure 1:**
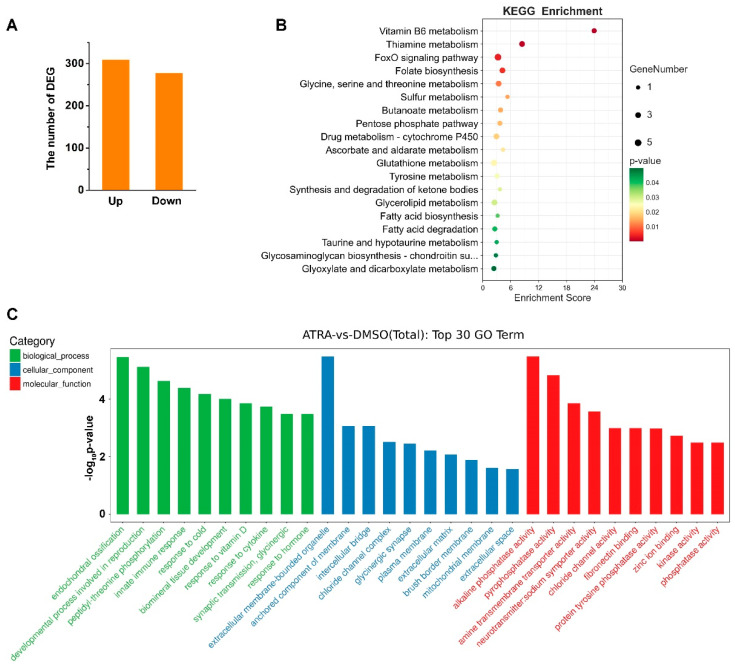
Transcriptome analysis of *C. gigas* after ATRA treatment. (**A**) Numbers of differentially expressed genes (DEGs) in *C. gigas* after all-trans retinoic acid (ATRA) treatment. Fold change ≥ 2, *p* values < 0.05. (**B**) KEGG pathways enriched for the ATRA-regulated genes. *p* values < 0.05. (**C**) GO enrichment analysis of DEGs in ATRA-treated oysters. *p* values < 0.05.

We previously identified the molecular characteristics of two RA receptors, *Cg*RAR and *Cg*RXR, in *C. gigas* [22]. To further study the genes that might be regulated by RA in *C. gigas*, transcriptome sequencing was carried out on samples collected from DMSO- and ATRA-treated individuals. A total of 586 DEGs were identified (fold change ≥ 2, *p* value < 0.05) between ATRA- and DMSO-treated groups (Table S1). Among them, 309 genes were upregulated and 277 were downregulated upon ATRA treatment (Figure 1A). KEGG pathway analysis showed that the 587 DEGs were mainly enriched in several metabolic related pathways, including Vitamin B6 metabolism, thiamine metabolism, glutathione metabolism, and so on (Figure 1B). GO enrichment analysis revealed that the DEGs were significantly enriched in endochondral ossification, the development process involved in reproduction, and innate immune response in the categories of biological process (Figure 1C). Taken together, these results suggested that ATRA is mainly involved in reproductive, developmental, immunity, and metabolic processes in *C. gigas*.

### 2.2. ATRA Induce Several TYR Genes Expression

**Figure 2:**
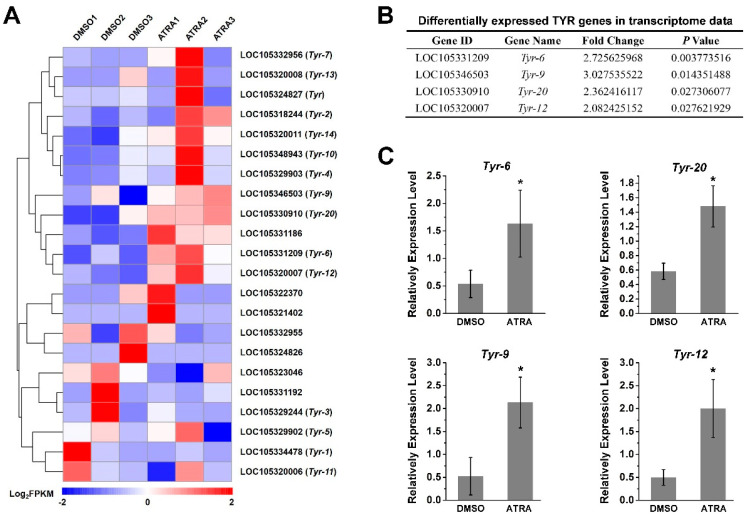
Expression of TYR gene after ATRA treatment. (**A**) The expression profiles of 22 TYR genes identified in transcriptome sequencing; (**B**) differentially expressed TYR genes in transcriptome data (fold change ≥ 2, *p* values < 0.05); (**C**) verification of TYR gene expression by RT-qPCR. Relative expression of TYR genes after ATRA treatment. Error bars represent means of three replicates ± SD (standard deviation, *n* = 6). (* *p* < 0.05; Student’s *t* test).

Of interest to us, the tyrosinase metabolic pathways were also enriched according to the result of the KEGG analysis (Figure 1B). It has been reported that RA contributes to melanogenesis and melanin production in vertebrates [34,35]. In order to explore the relationship between RA pathway and melanin production in *C. gigas*, we screened the expression of tyrosinase (TYR) genes, the key genes for melanin production, from transcriptome data. A total of 22 TYR genes were identified (Figure 2A and Table S2), of which the expression of four *TYR* genes, including *Tyr-6* (LOC105331209), *Tyr-9* (LOC105346503), *Tyr-20* (LOC105330910), and *Tyr-12* (LOC105320007) were differentially expressed (fold change ≥ 2, *p* value < 0.05) in the ATRA-treated group compared with the DMSO-treated group (Figure 2B). RT-qPCR analysis was performed to verify the RNA sequencing data. The result showed that expression of *Tyr-6*, *Tyr-9*, *Tyr-20*, and *Tyr-12* increased significantly after ATRA injection (Figure 2). We hypothesized that RA affects the expression of TYR genes, and therefore affects melanin production in *C. gigas*.

**Figure 3:**
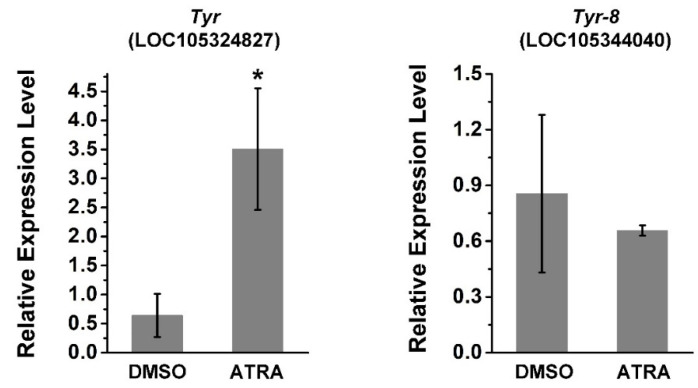
ATRA upregulates the expression of *Tyr* and *Tyr*-*8***.** Error bars represent means of three replicates ± SD (standard deviation, *n* = 6, * *p* < 0.05; Student’s *t* test).

*Tyr* (LOC105324827) and *Tyr-8* (*Trp2*, LOC105344040) are tyrosinase genes related to melanin production in *C. gigas* [36,37]. Thus, the relative expression of these two genes was investigated using RT-qPCR after ATRA treatment. As shown in Figure 3, the expression of *Tyr* was significantly upregulated by ATRA treatment, whereas there was no significant change in the expression of *Tyr-8* after ATRA treatment. This suggests that the RA pathway may regulate expression of certain TYR genes in *C. gigas*.

### 2.3. CgRAR Combines with RARE

**Figure 4:**
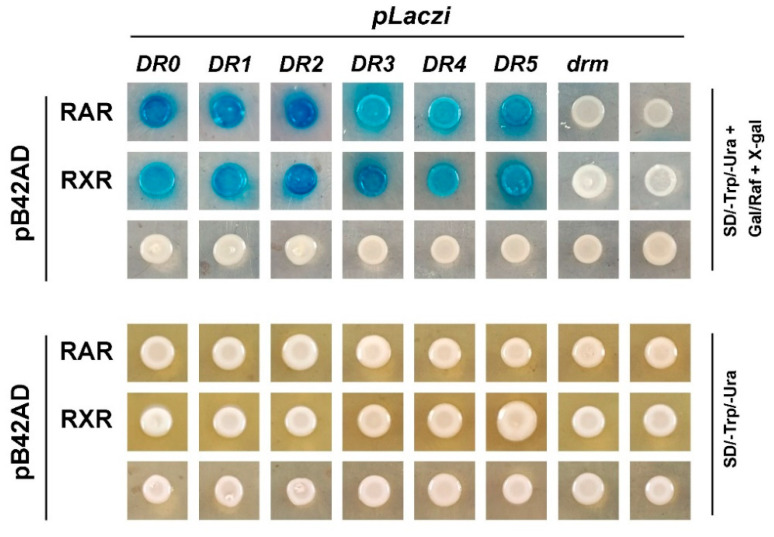
*Cg*RAR and *Cg*RXR bind *DR0–DR5* in yeast. Both *Cg*RAR and *Cg*RXR can successfully bind the artificial triple repeated *DR0*–*DR5* sequences in yeast cells, whereas it did not bind to the *drm* sequence. Yeast stains co-transferred with fusion plasmids were grown on SD/-Trp/-Ura select medium (**lower** rows) and were transferred to SD/-Trp/-Ura select medium with Gal/Raf and X-gal (**upper** rows) to detect the activation of *LacZ* reporter gene.

RAR and RXR are members of the nuclear receptor superfamily, which directly bind to the promoter regions of target genes to regulate their expression. Our previous study revealed that both *Cg*RAR-BD and *Cg*RXR-BD fusion vectors exhibited strong self-activation when co-transferred with *the pGAD T7* vector in the yeast two-hybrid assay [22]. This suggests that *Cg*RAR and *Cg*RXR may have transcriptional activities. Thus, to detect the binding activity of *Cg*RAR and *Cg*RXR to the DNA sequence, a yeast one-hybrid assay was performed to investigate whether *Cg*RAR or *Cg*RXR interacts with DRs consisting of the (A/G)G(G/T)TCA core sequence. *DR0*–*DR5*, containing the RARE core sequence “AGGTCA” separated by 0–5 nucleotides, was produced and inserted into *the pLacZi* reporter vector to generate a fusion plasmid. *CgRAR* and *CgRXR* were then fused to the *pB42AD* vector. The mutated *drm*, produced by random mutation of the RARE core sequence “AGTTCA”, was used as a negative control. Yeast stains co-transferred with *pB42AD* and *pLacZi* empty vectors were used as blank controls. Yeast stains co-transferred with *Cg*RAR and *DR0*–*DR5* showed obvious positive reactions on a chromogenic medium (Figure 4). *Cg*RXR also showed binding affinity to *DR0*–*DR5*, but not to *drm* (Figure 4). These results indicate that both *Cg*RAR and *Cg*RXR can combine with *DR0*–*DR5* in yeast.

### 2.4. CgRAR and CgRXR Bind the RARE Motif in the Promoter of TYR Genes

**Figure 5:**
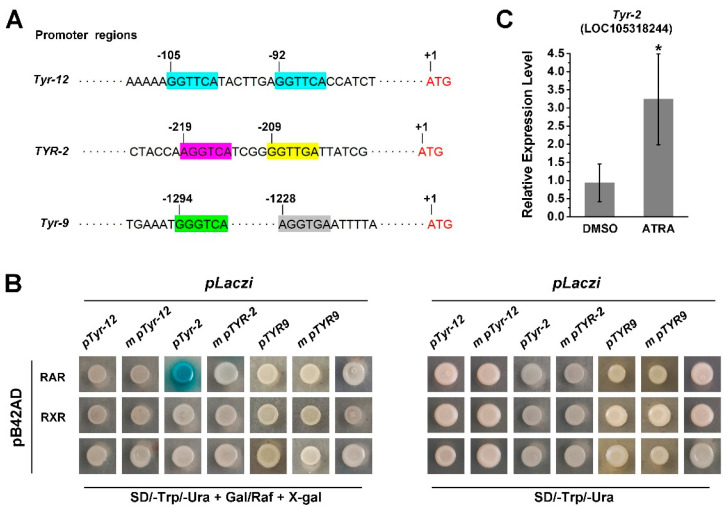
*Cg*RAR bind the promoter of *Tyr-2* in yeast. (**A**) Schematic structure of RAREs in the TYR promoters. (A/G)G(G/T)(G/T)(G/C)A core sequence are marked in colored frames, and the numbers of scale plates indicate the location of these core sequence upstream from the ATG start codon; (**B**) *Cg*RAR combine with *Tyr-2* promoter; (**C**) relative expression level of *Tyr-2* upon ATRA treatment. Error bars represent ± SD (*n* = 6, * *p* < 0.05; Student’s *t* test).

Since RA treatment activates the expression of several TYR genes and RA receptors of the Pacific oyster and these have the ability to bind RARE, we further investigated the role of *Cg*RAR and *Cg*RXR in the regulation of TYR gene expression. First, we screened for the RARE motif in the promoter regions of *C. gigas* TYR genes. A potential RARE, a DR consisting of two “GGTTCA” spaced by seven nucleotides was found in *Tyr-12* and a DR consist of (A/G)G(G/T)T(G/C)A core sequence spaced by four nucleotides was found in the promoter region of *Tyr-2* (LOC108318244) (Figure 5A). To elucidate whether *Cg*RAR and *Cg*RXR would interact with these potential RAREs in the promoter region of these oyster tyrosinase genes, the 222 bp fragment from the promoter of *Tyr-12* (*pTyr-12*) and 121 bp fragment from the promoter of *Tyr-2* (*pTyr-2*) were amplified. Both *pTyr-12* and *pTyr-2* contained the potential RARE motifs, and the fragments were fused with the *pLacZi* vector, respectively, for yeast one-hybrid assays. The same fragments with mutant RARE core sequences (*m pTyr-12* and *m pTyr-2*) were used as negative controls. The Y1H assay revealed that *Cg*RAR interacted with the RARE present in the *Tyr-2* promoter, whereas no binding activity of *Cg*RAR to *pTyr-12* was detected in yeast (Figure 5B). A mutation of the “AGGTCA” core sequence in the *Tyr-2* promoter abolished the *LacZ* reporter gene activation by *Cg*RAR (Figure 5B). Furthermore, it failed to detect the binding of *Cg*RXR to *pTyr-12* or *pTyr-2* by Y1H. Taken together, these results illustrate that *Cg*RAR binds the promoter of *Tyr-2*, and this depends on the DR4 consisting of “AGGTCA” and “CCTTGA” separated by four base pairs. The RT-qPCR was also carried out to detect whether ATRA affects the expression of *Tyr-2*, and the result reveals an upregulation of *Tyr-2* by ATRA treatment (Figure 5C).

Two (A/G)G(G/T)T(G/C)A core sequences were found in the promoter of *Tyr-9*. These were separated by 58 bases and did not form short DR motifs (Figure 5A). RT-qPCR analysis also revealed that *Tyr-9* was upregulated by ATRA treatment (Figure 2). Thus, we wonder whether *Tyr-9* could be directly regulated by the *C. gigas* RA receptor. A 173 bp promoter region of *Tyr-9* (*pTyr-9*) was amplified and constructed into the *pLacZi* vector. Y1H results showed that the LacZ reporter gene could not be activated by *Cg*RAR or *Cg*RXR, suggesting that neither *Cg*RAR nor *Cg*RXR binds to this fragment in the *Tyr-9* promoter (Figure 5B).

## 3. Discussion

### 3.1. Biological Roles of RA in C. gigas

RA is an important metabolic product derived from vitamin A that functions as a signaling molecule in numerous animals. RA participates in the regulation of many biological processes, including growth and development, reproduction, differentiation, and apoptosis. In invertebrates RA contributes to several processes, including the formation and modulation of central synapses, neuronal differentiation, and shell development [9,10,38]. RA also can affect calcium signaling in the neurons of adult mollusks [39]. Here, we injected ATRA into the adductor muscle of *C. gigas* to investigate the role of RA in mollusks. Mantle tissue samples were collected for transcriptomic sequencing, and this showed that the expression of 586 genes were affected by ATRA treatment, with 309 upregulated and 277 downregulated genes. GO clustering and KEGG pathway analysis showed that the DEGs were mainly related to development, metabolism, reproduction, and immunity (Figure 1B,C). Other studies in mammals have also shown that RA is associated with mucosal non-specific immunity and can promote antibody production in specific immunity [40,41]. Therefore, RA in *C. gigas* mainly participate in the biological processes of reproduction, development, and immunity, which is similar to those of mammals.

### 3.2. The Binding Ability of CgRAR/CgRXR to RAREs and TYR Gene Promoter

As a ligand, RA regulates the expression of different genes through RA receptors. RAR and RXR are two classes of RA receptors belonging to the nuclear receptor superfamily. After the perception of RA, RAR and RXR interact with each other to form heterodimers or interact with themselves to form homodimers [8,14]. RAR and RXR directly bind to specific sequences of target genes and regulate target gene expression following a heterodimer or homodimer formation [5].

Our previous study revealed that the expression of both *Cg*RAR and *Cg*RXR was upregulated upon ATRA treatment [22]. In this study, we found that the expression of four TYR genes were upregulated after ATRA treatment according to the transcriptome data (Figure 2A,B). This result was subsequently conformed by RT-qPCR (Figure 2). The specific binding sites of RAR or RXR in the promoter regions of target genes are mostly RAREs composed of DRs spaced by 0–5 base pairs. Our results also revealed that *Cg*RAR and *Cg*RXR bind DR0–DR5 sequences in yeast (Figure 4). The interaction between *Cg*RXR and DRs has been confirmed using an electrophoresis mobility shift assay (EMSA) [25]. Two possible RAREs were identified in the promoter regions of *Tyr-12* and *Tyr-2* (Figure 5A). Therefore, we hypothesized that *Cg*RAR and *Cg*RXR can directly bind to the promoters of these TYR genes. The results of the Y1H assay suggest that *Cg*RAR can directly bind to the DR4 consisting of two core sequences, “AGGTCA” and “GGTTGA”, separated by four nucleotides in the *Tyr-2* promoter (Figure 5B). Thus, we conclude that *Cg*RAR can directly bind to the promoter region of *Tyr-2*. ATRA treatment activates the expression of *Tyr-2*. Further studies are needed to determine whether the binding of *Cg*RAR to the *Tyr-2* promoter in vitro directly contributes to the activation of *Tyr-2* expression.

Previous studies have reported that RAR may not bind RA directly in some mollusks [20,21]. In *Thaisb clavigera*, *Acanthochitona crinite*, *Patella vulgate*, *N. lapillus*, and *C. gigas*, RAR does not activate the transcription of reporter genes in response to stimulation by retinoids [20,42]. In vertebrates, RAR-RXR binds to RAREs in the absence of ligands, whereby unliganded RARs recruit transcriptional co-repressors to negatively regulate the transcription of target genes [43]. One possibility is that in *C. gigas*, RAR binds to the promoter region to recruit co-repressor factors to inhibit the expression of target genes in the absence of RA. After RA treatment, RAR no longer binds to RARE and dissociates from the promoter region relieving inhibition, and allowing the expression of target genes to reactivate.

Although the expression of *Tyr-12* and *Tyr-9* was also activated after ATRA treatment (Figure 2), and a DR consisting of two core sequence “GGTTCA” spaced by seven bases was found in the *Tyr-12* promoter region, we did not detect the binding activity of *Cg*RAR or *Cg*RXR to the promoter regions of these two genes. Furthermore, the expression of *Tyr*, *Tyr-6*, *Tyr-9*, *Tyr-12,* and *Tyr-20* was upregulated by ATRA treatment; although typical RARE, as defined in vertebrates, was not found in the promoters of these genes. In vertebrates, hundreds of genes have been found to be RA-inducible, but only around 20 have been verified to contain functional RAREs [5]. Except those RA receptors directly binding to target gene promotors to regulate their expression, some target genes can be indirectly expressed by other molecular mechanisms in response to RA [5]. Thus, we suspect that in *C. gigas*, ATRA activates the expression of these TYR genes in a different manner, other than directly binding to the promoter region of these TYR genes by RA receptor.

### 3.3. The Relationship between RA Pathway, TYR Genes, and Melanin

We previously compared transcriptomes of black- and white-shelled oyster mantle tissue, and found that expression of the retinaldehyde dehydrogenase gene (which catalyzes the production of RA), as well as the tyrosinase gene of black-shelled oysters was significantly elevated relative to white-shelled oysters [37]. In this study, we found that the expression of *Tyr*, *Tyr-6*, *Tyr-9*, *Tyr-12,* and *Tyr-20* was elevated after ATRA treatment (Figure 2 and Figure 3). Tyrosinase, encoded by the *Tyr* gene, is a multifunctional copper-containing oxidase that catalyzes the first two steps of melanin production in mammals, and is found in most organisms other than viruses. An expansion of tyrosinase genes has been observed in many bivalves, with more than 26 *Tyr* genes found in *C. gigas* [30,44]. In *C. gigas*, the expression of *Tyr-9* and *Tyr-12* in black-shelled oyster were significantly higher than that in white-shelled oysters [37]. A recent study revealed that *Tyr* was strongly expressed in black-shelled oysters, whereas almost no expression was detected in white-shelled oysters [36]. Several studies have linked RA with melanin production in vertebrates. RA influences melanocyte differentiation and proliferation in a dose- and time-dependent manner [45]. In mouse B16F10 melanoma cells, RA increases melanogenesis [34]. In chick RPF cells, RA induces TGF-β, which inhibits RPE cell proliferation and induces melanin synthesis [35]. Therefore, it is possible that the ATRA-induced upregulation of TYR genes contributes to melanin production in *C. gigas*. In addition, the role that *Tyr-6* and *Tyr-20* plays in melanin production requires further investigation.

## 4. Material and Methods

### 4.1. Experimental Materials

Adult oysters used in this study were collected from a local farm in Yantai, China. Oyster individuals with an average height of 60 mm and average weight of 24.6 g were selected for further studies. In laboratory conditions, all of the selected oysters were acclimated in filtered seawater for 7 days at temperatures of 19–22 °C under aerated conditions before the experiment. During cultivation, the oysters were fed with *Isochrysis galbana* twice a day and the seawater was changed daily.

### 4.2. RA Treatment

RA treatment was performed as previously described with a slight modification [22]. Briefly, ATRA (Sigma-aldrich) dissolved in DMSO was injected into the *C. gigas* individuals via the adductor muscle with a final concentration of 10 μM. Oysters injected with DMSO were used as the control. Injections were carried out every two days for a total of eight days. After treatment, mantle tissues were collected and frozen with liquid nitrogen for transcriptomic sequencing or RT-qPCR analysis.

### 4.3. Transcriptome Sequencing Analysis and Bioinformatics Analysis

For transcriptome sequencing, 18 individuals were randomly divided into two groups and injected with ATRA and DMSO, respectively. After injection, the mantle tissues of three oyster individuals were weighed in equal quantities and mixed to form one sample, and each group contained three sample replicates. Transcriptome sequencing was performed using the Illumina NovaSeq 6000 PE150 platform at OE Biotech Co., Ltd., (Shanghai, China). More than 46 million clean reads were obtained for each sample and mapped to the reference genome of *C. gigas* (GCF_902806645.1). The DEGs were identified using the DESeq functions estimateSizeFactors and nbinomTest [46], with a fold change > 2 and *p* value < 0.05. Gene ontology (GO) enrichment and Kyoto Encyclopedia of Genes and Genomes (KEGG) [47] pathway enrichment analysis of DEGs was performed using R based on hypergeometric distribution.

### 4.4. RNA Extraction and RT-qPCR

For RT-qPCR analysis, 12 individuals were randomly divided into two groups and, after injection, the mantle tissue RNA was isolated using TRIzol reagent (Invitrogen, USA) according to the manufacturer’s protocol. cDNA was synthesized using the PrimeScript™ RT Master Mix kit (TaKaRa, Japan), and RT-qPCR analysis was performed using SYBR Premix Ex Taq II (TaKaRa, Japan) and a *Bio-rad* CFX connect PCR instrument. The *RS18* gene was used as the reference gene for internal normalization. Relative expression levels of the target genes were calculated with the 2^−△△CT^ method. Primers used for RT-qPCR are listed in Table S3.

### 4.5. Yeast One-Hybrid (Y1H) Assay

The DNA-binding activity of RA receptors in *C. gigas* was determined using the typical RARE core sequence as defined in vertebrates. DR0–DR5, formed by triple tandem copies of the RARE core sequence (AGGTCA) separated by 0–5 nucleotides, were produced by primers annealing. The generated DR0–DR5 sequences were then inserted into the *pLacZi* vector to construct the reporter plasmids. The mutated RARE core sequence was used to form mutated DR (*drm*) as a negative control. The primers used for the fusion vector construction are listed in Table S4.

To examine the interaction between *Cg*RAR/*Cg*RXR and TYR *gene* promoters, several 100–250 bp fragments from the promoter regions of the TYR genes containing the RAREs were amplified and inserted into the *pLacZi* vector. The RARE core sequences were mutated and used as negative controls.

Full-length coding sequences of *Cg*RAR and *Cg*RXR were cloned into the *pB42AD* vector, respectively. The yeast strain EGY48 was used to assess protein–DNA interactions in this study. Yeast co-transformants were screened on a selective SD/-Trp/-Ura medium, and positive transformants were screened using a SD/-Trp/-Ura chromogenic medium with Gal/Raf and X-gal.

## 5. Conclusions

Our results show that ATRA may participate in the biological processes of development, metabolism, reproduction, and immunity in oysters, which are similar to those in mammals. Expression of several melanin-related TYR genes were upregulated by ATRA. The RA receptors *Cg*RAR and *Cg*RXR can bind RAREs, and *Cg*RAR interacts with the DR sequence in the promoter of *Tyr-2*. The expression of six TYR genes (*Tyr-6*, *Tyr-9*, *Tyr-12*, *Tyr-20*, *Tyr*, and *Tyr-2*) were upregulated by ATRA, whereas only the *Tyr-2* promoter could be activated by the CgRAR, indicating that ATRA activates the expression of these TYR genes in a different manner. Thus, through the classical RA mechanism, *Cg*RAR directly binds to the promoter region of *Tyr-2*, and then regulates the transcriptional expression of *Tyr-2* in response to ATRA treatment. In addition, ATRA may activate TYR gene expression in other ways without directly binding to the promoters of these target genes by RA receptors.

## Data Availability

The data of current study are available from the corresponding author on reasonable request.

## Supplementary Materials

The following supporting information can be downloaded at: https://www.mdpi.com/article/10.3390/ijms232112840/s1. Table S1. Differently expressed genes identified between ATRA- and DMSO-treated groups. Table S2. The expression profiles of TYR genes in transcriptome data. Table S3. Primers used for RT-qPCR analysis. Table S4. Primers used for fusion vectors construction.

## Abbreviations

retinoic acid	RA
retinoic acid receptor	RAR
retinoid x receptor	RXR
retinoic acid response element	RARE
all-trans retinoic acid	ATRA
differentially expressed genes	DEGs
yeast one-hybrid	Y1H
